# Label-Free and Reproducible Chemical Sensor Using the Vertical-Fluid-Array Induced Optical Fiber Long Period Grating (VIOLIN)

**DOI:** 10.3390/s20123415

**Published:** 2020-06-17

**Authors:** Deming Hu, Zhiyuan Xu, Junqiu Long, Peng Xiao, Lili Liang, Lipeng Sun, Hao Liang, Yang Ran, Bai-Ou Guan

**Affiliations:** Guangdong Provincial Key Laboratory of Optical Fiber Sensing and Communications, Institute of Photonics Technology, Jinan University, Guangzhou 510632, China; hdming@stu2017.jnu.edu.cn (D.H.); 2016jnuxzy@stu2018.jnu.edu.cn (Z.X.); long@stu2018.jnu.edu.cn (J.L.); xiaopeng@stu2017.jnu.edu.cn (P.X.); lianglili@jnu.edu.cn (L.L.); lpsun@jnu.edu.cn (L.S.); tlianghao@jnu.edu.cn (H.L.); tguanbo@jnu.edu.cn (B.-O.G.)

**Keywords:** fiber optics, long period grating, fiber optical sensors, refractive index, chemical sensing, mercapto compound

## Abstract

Fiber optical refractometers have gained a substantial reputation in biological and chemical sensing domain regarding their label-free and remote-operation working mode. However, the practical breakthrough of the fiber optical bio/chemosensor is impeded by a lack of reconfigurability as well as the explicitness of the determination between bulk and surface refractive indices. In this letter, we further implement the highly flexible and reproducible long period grating called “VIOLIN” in chemical sensing area for the demonstration of moving those obstacles. In this configuration, the liquid is not only leveraged as the chemical carrier but also the periodic modulation of the optical fiber to facilitate the resonant signal. The thiol compound that is adsorbed by the fluidic substrate can be transduced to the pure alteration of the bulk refractive index of the liquid, which can be sensitively perceived by the resonant drift. Taking advantage of its freely dismantled feature, the VIOLIN sensor enables flexible reproduction and high throughput detection, yielding a new vision to the fiber optic biochemical sensing field.

## 1. Introduction

Optical fiber refractometer is a fast-developing candidate for biological and chemical sensing due to its promising feature of without being subjected to molecular dying-process and laboratory settings [1,2,3,4]. Various types of fiber optic refractive index sensors have been developed to pry about the ambient medium and can be categorized into gratings [5,6,7], interferometers [8,9,10], and resonators [11,12,13,14]. Among those sensors, long-period fiber grating (LPG) guides the core lightwave to outer cladding region via a longitudinal index modulation structure with a period of hundreds of microns to millimeters, allowing light to interplay further with the ambient medium. The LPG outperforms its counterparts lying in the combination of ease of fabrication, the flexibility of design, the abundance of serviceable signals, cost-effectiveness, and high sensitivity [15,16,17]. As a consequence, the LPG refractometer is soon considered as a competitive approach for label-free and in-situ assessing biological and chemical targets in which the molecular reaction events could be transduced simply by the grating resonance shift.

In the biosensing field, Chiavaioli et al. reported an IgG/Anti-IgG bioassay using the LPG with a copolymer functional layer [18]; Liu et al. utilized the graphene oxide functionalized LPG for the detection of the IgG [19] and hemoglobin [20], respectively; Xiao et al. facilitated a higher order diffraction LPG for the analysis of prostate specific antigen [21]; Piestrzyńska et al. proposed a tantalum oxide nano coated LPG for the test of avidin and *Escherichia coli* [22]; Yang et al. used a polyelectrolyte coated LPG to sense the *staphylococcus aureus* bacteria [23]; Janczuk-Richter et al. achieved the LPG-based the virus sensor [24] and Quero et al. realized the cancer biomarker detection upon applying the reflection mode of the LPG [25].

In chemosensing area, Yin et al. presented a 3D patterning of poly(acrylic acid) ionic hydrogel decorated LPG pH Sensor [26]; Wang et al. adopted LPG sensing approach to measure humidity [27]; Wang et al. carried out an LPG sensor with nano-assembled porphyrin layers to detect the concentration of ammonia gas [28]; Hsu et al. used a double notched LPG to sense CO_2_ gas [29]; Baliyan et al. proposed a lipid sensor using the LPG [30]; Celebanska et al. and Tripathi et al. had conducted their trials on the aptasensor using LPG for monitoring cocaine [31] and toxin [32], respectively. 

For those demonstrations, imposing a permanent index texture to the fiber is considered as a laboratory routine to make an LPG and the analytical solution commonly serves as the bulk milieu. In this strategy, reconfigurability and recyclability of the LPG sensor, however, remain challenging once the target molecules are immobilized specifically on the device, impeding the promotion of the LPG biochemical sensor with respect to the scenarios of high throughput screening as well as commercial Point-of-Care test (PoCT). 

To overcome the limitations, a promising alternative was reported by our group through the use of a microfiber for the readout of information of a periodically patterned liquid, which can be described in principle as “Liquid renders the resonance to light. Light deciphers the secret of liquid” [33]. The vertical-fluid-array-induced optical microfiber long-period grating, analogous to a violin instrument, further harnesses the light-liquid interaction in a highly flexible and practical manner and facilitates the reproducibility through its nature of free combination of the two components, i.e., “fiber bow” and “fluidic pad”. 

In this letter, we further develop and demonstrate the VIOLIN sensor for the determination of chemicals. Mercapto compounds, including cysteine, homocysteine, and glutathione, play an essential role in the daily activity of lives. The alteration of the level of the mercapto compounds in bio-system may forecast some critical diseases, such as cancer or cardiovascular disease [34,35,36,37,38]. Here, a kind of mercapto compound, *p*-mercapto benzoic acid (*p*-MBA), was involved in the test. The channels of the VIOLIN, which are gilded by a thin gold layer, could adsorb the *p*-MBA molecules as the solution flows in the channels, resulting in a decrease of the bulk refractive index of the liquid and therefore a blueshift of the VIOLIN resonance. The amount of the resonant blueshift indicates the concentration of the *p*-MBA of the liquid quantitatively. Compared with the state-of-the-art of the LPG sensors in literature, this work fully takes advantage of the liquid-light interaction and the recombined feature of the VIOLIN structure. With high specificity and reconfigurability, this novel VIOLIN sensor would bear great potential and occupy a competitive niche in the label-free detection of chemical and biological molecules. 

## 2. Materials and Methods

### 2.1. Materials

The optical fiber we used is the commercial single-mode telecom silica fiber (8/125 μm) with a numerical aperture (N.A.) of 0.14, which was obtained from Corning Inc (SMF-28, Corning, NY, USA). Rhodamine B (RhB) and p-Mercaptobenzoic acid (*pMBA*) were purchased from Macklin (Shanghai, China) and Aladdin (Shanghai, China), respectively. All the chemicals and reagents are at the highest purity grade available and used as received. The polymethyl methacrylate (PMMA) plate was gotten from Xintao Group (Shenzhen, China). The pure 100%-ethanol was acquired from HUSHI (Shanghai, China) and was used throughout the experiment.

### 2.2. Instrumentation

The CO_2_ laser was purchased from SYNRAD with a home-made lasing-manipulation system (SYNRAD 48, Mukilteo, WA, USA). The gold-sputtering machine was the Ion Sputter Coater (SBC-12, KYKY Ltd., Beijing, China). A broadband LED source (GoLigtht Ltd., Shenzhen, China) was utilized to launch a continuous spectrum light ranging from 1250 to 1650 nm into the microfiber. An optical spectrum analyzer (OSA, AQ6370D, YOKOGAWA, Tokyo, Japan) was used for monitoring the output spectrum of the microfiber. A standard refractometer (PAL-RI, ATAGO, Tokyo, Japan) was used to calibrate the refractive index of the solution. A scanning electronic microscope (SEM Phenom pure+, Thermo Fisher Scientific, Eindhoven, The Netherlands) and a Raman Spectrometer (DXR3, Thermo Fisher Scientific, Waltham, MA, USA) were employed to characterize the substrates.

### 2.3. Principle of VIOLIN Device

The mode characteristics of an optical fiber could be fully elucidated through the finite-element-method (FEM) to solve a three-layer cylindrical waveguide with the step-change indices. For an LPG, the coupling between the fundamental mode and higher-order mode can be described according to the phase match condition as [39]:

(1) $$\left|\beta 1-\beta 2\right|=\frac{2\pi }{\Lambda }$$

where *β*1 and *β*2 are the propagating constants of the fundamental mode and one higher-order mode, respectively, and Λ refers to the grating period. The resonant wavelengths (*λ*_res_) of an LPG can be expressed by transforming the Equation (1) to:

(2) $$\lambda_{\mathrm{res}}=\left(N_{eff_{\text{\_}o}}-N_{eff{\text{\_}}_{v}}\right)\times \Lambda =\Delta n\times \Lambda$$

where *N_eff_o_* and *N_eff_v_* represent the effective indices of the fundamental mode and the v-order mode, respectively. If the pattern of the grooves has a duty-cycle (Λ) of 1100 μm, a microfiber with a diameter of 35 μm is optimally selected for the design of an appropriate resonant wavelength within the telecommunication waveband. The inter-modal coupling occurs between the modes of LP01 and LP21 [33].

### 2.4. Configuration of the VIOLIN Sensor

**Figure 1:**
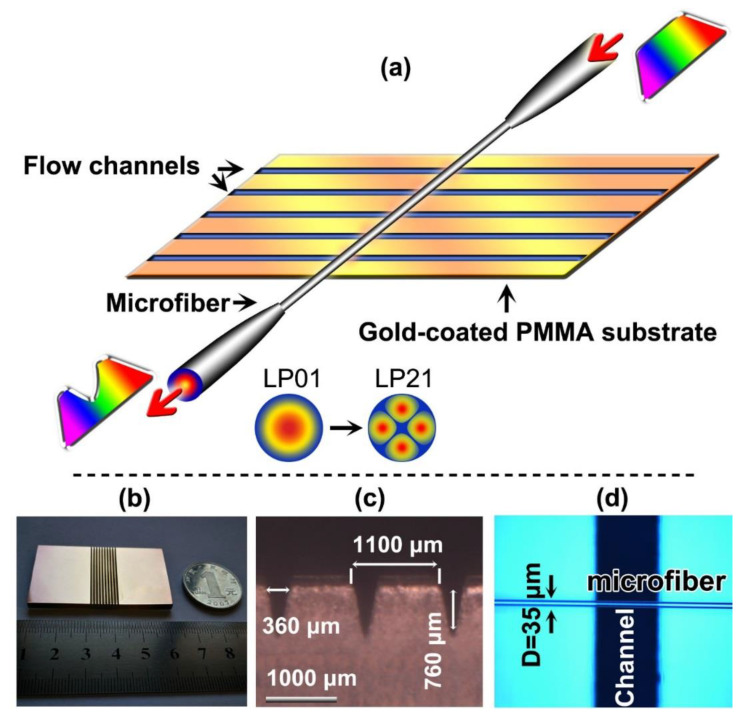
(**a**) Diagrams of the schematic of the VIOLIN sensor; Inset: the coupling between the two modes as indicated. (**b**) Real product of the substrate. (**c**) Scanning electron microscopic (SEM) image of the fluidic channel (horizontal view). (**d**) The optical microscopic image of the microfiber vertically placed on the substrate (perpendicular view).

Figure 1a shows the diagram of the VIOLIN structure, which is consist of two elements, “string pad”—The substrate and “bow”—The optical microfiber. First, the substrate of the VIOLIN takes the PMMA plate as the base that has dimensions of (50 × 30 × 3) mm^3^ (L × W × H). The 10.6 μm—Wavelength pulsed CO_2_ laser is used to inscribe the V-groove-channels on the PMMA plate. The laser beam is focused to a spot of a diameter of 50 μm by a ZnSe lens. The scanning speed, output power, and repetition frequency of the CO_2_ laser are set to 1000 mm/s, 25 W, and 8 kHz, respectively, which are precisely controlled by the home-made control system. Ten channels are neatly designed, as shown in Figure 1b. Each V-groove-channel is engraved through an 8 cycle/3 s -laser irradiation. The channel has a width of 360 μm and a depth of 760 μm, as seen in Figure 1c. The period of the channels is 1100 μm. After the inscription, the substrate is coated with a thin film of gold by the Ion Sputter Coater. Second, the optical microfiber is drawn from the commercial single-mode fiber using the flame-heated drawing technique [40,41]. The diameter of the taper waist of the microfiber is set to 35 μm and placed vertically to the V-groove-channel array, as shown in Figure 1d. The analytical liquids are pipetted into the channels for the activation of the VIOLIN. The broadband LED and OSA are connected with the microfiber by the two ends to monitor the transmission spectrum and thus facilitate the analytical readout. The experiment is carried out in the air-condition controlled lab and the environmental temperature is kept at 24 °C. We have tried to make all the experiments at almost the same room-temperature to eliminate the temperature cross-sensitivity [33].

## 3. Results

### 3.1. Refractive-Index Response of the VIOLIN

**Figure 2:**
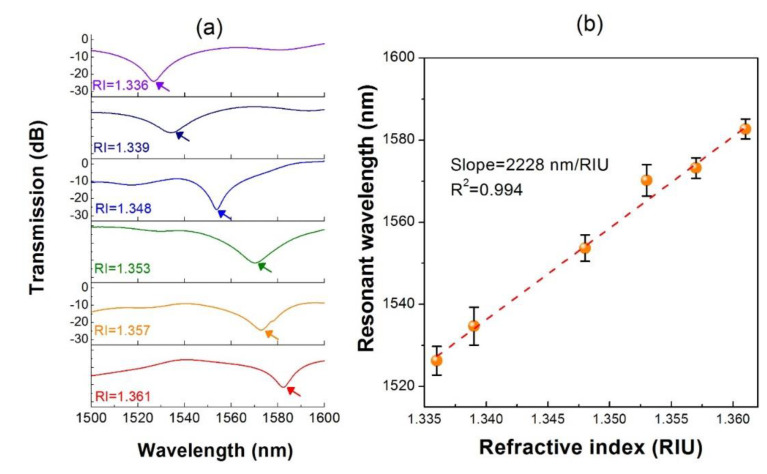
(**a**) Spectral change of the VIOLIN with respect to the RI increase of the liquid. (**b**) Response curve between the resonance wavelength and the liquid RI. Error bars indicate the standard deviations of three independent measurements and below were the same.

The label-free sensing often harnesses a suitable and reliable transducing parameter. In this configuration, since the liquid plays an indispensable role in the VIOLIN structure, solutes in the liquid would affect the resonant spectrum tremendously through the alteration of the refractive index. As a consequence, investigating the influence of changing different RI of liquids in the grooves is of great necessity. A series of liquids are prepared by mixing the pure ethanol and deionized water with different ratios. The concentrations of the ethanol in the mix were adjusted and represented by the medium RI. The RIs of the mix were calibrated by the refractometer, ranging from 1.336 to 1.361. Figure 2a displays the spectra of the resonances with respect to those liquids which are pipetted into the channels. As the RI of the fluid increases, the resonant wavelength moves to the longer wavelength. It is worth noting that variation of the spectral shape could be observed in the RI increasing process. The reason should be attributed to that the liquid was not flowing ideally in the groove channels with identical volume through manual operation of pipetting, probably leading to the insufficient interaction between the liquid and the microfiber at some channels. Nevertheless, the periodic structure could still guarantee a convincing resonant wavelength of the configuration for revealing the RI response. The response curve, with a linear correlation (R^2^ = 0.99), outputs a sensitivity of ~2228 nm/RIU, which is similar to our previous report. The high RI sensitivity allows the VIOLIN to perceive a slight change in the concentration of the chemical molecules in the solution.

### 3.2. VIOLIN for Mercapto-Group Chemical Sensing

**Figure 3:**
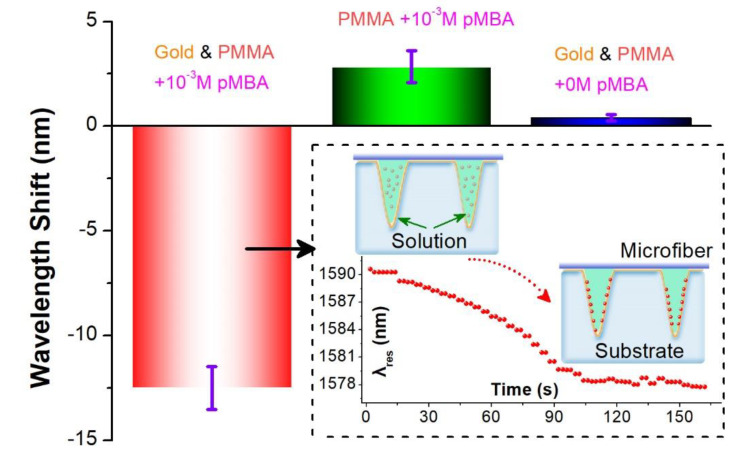
Response of the VIOLIN sensors for targeting *pMBA* solutions. Inset: the diagram and dynamic process of *pMBA* sensing using the gilded-VIOLIN.

Since chemicals with mercapto-group tend to be adsorbed covalently to the metal surface [42,43,44], the VIOLIN can be utilized as the thiol group chemical sensors by virtue of the gold-gilded substrate. In this experiment, we employ the p-mercapto benzoic acid (*p*MBA) as the chemical which is dissolved into the pure ethanol with the concentration of 10^−3^ M. As the liquid is pipetted into the channels, the gold layer at the wall of the channel captures the *p*MBA molecules from the solution, leading to a lower density of the solution. Therefore, the resonance dip of the VIOLIN exhibits a blue-shifting curve along with time elapsing (150 s) due to the refractive index decrease during the adsorption process, as shown in the inset of Figure 3. The total wavelength shift is ~−12.5 nm, which can be obtained by the value subtraction between the plateaus from the end and the beginning. However, if the pure ethanol liquid without *p*MBA molecule is injected into the channels, the resonance maintains its spectral position as a reliable refractometer. By contrast, we test another VIOLIN without gilding the substrate to target the same *p*MBA solution. It can be seen in the Figure 3, gilding-free VIOLIN presents a significantly different response. The red-shift of the resonance probably indicates the weak binding between the *p*MBA and silica fiber via van der Waals force. Therefore, the gold layer in the channels of the VIOLIN is essential to facilitate mercapto-group chemical sensing.

To quantitatively investigate the sensing capability of the VIOLIN towards mercapto-group chemicals, we have prepared a series of concentrations of *p*MBA ethanol solutions ranging from 10^−5^ to 10^−1^ M. The liquids are pipetted into the channels in the sequential order of the concentrations from low to high. At each concentration, three solution samples are tested. The wavelength shift of the resonance (Δλ) is obtained by the subtraction between the wavelength values recorded at 120 s and the beginning of the process, respectively. 

**Figure 4:**
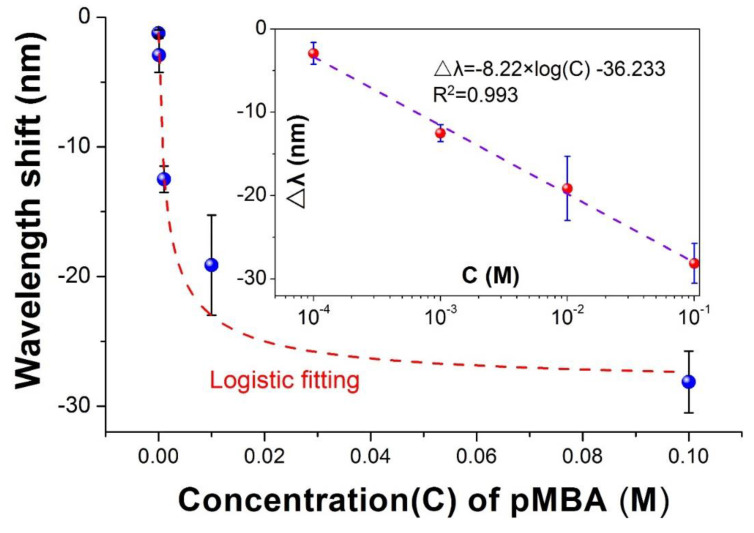
Response curve of sensing *p*MBA solutions with different concentrations using VIOLIN chemical sensor. Inset: the log-linear correlation at part region of the concentrations.

As illustrated in the Figure 4, the higher concentration of *p*MBA ethanol solution induces a larger response of the VIOLIN resonance as a result of more molecules are expelled from the solution and thus a more significant change of the refractive index. The curve follows a logistic fitting which is described as:

(3) $$\Delta \lambda =-28.49+\frac{27.85}{{\left(1+C/0.0016\right)}^{0.763}}\;,\;\left(R^{2}=0.976\right)$$

where *C* denotes the concentration of the *p*MBA. Moreover, a log-linear responding region was found with the range from 10^−4^ to 10^−1^ M. The relationship, shown in the inset of Figure 4, could be represented as

(4) $$\Delta \lambda =-8.22\times log(C)-36.233\;,\;\left(R^{2}=0.993\right)$$

**Figure 5:**
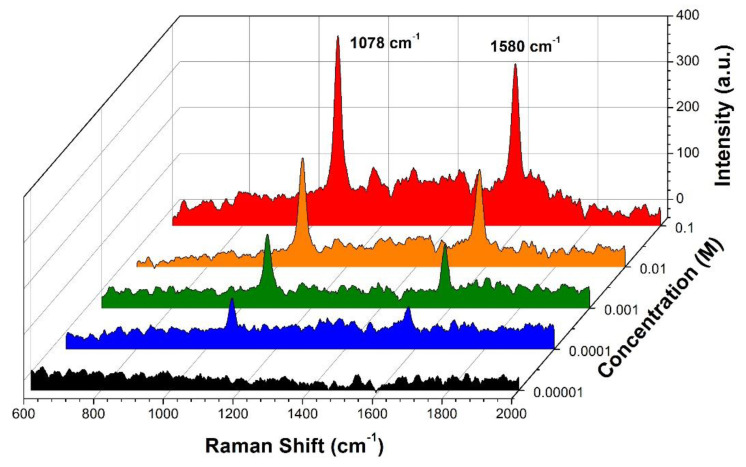
Raman intensities of the dried groove channel after the flowing of the *pMBA* ethanol solutions.

To verify the adsorption of the *p*MBA on the surface of the groove, we use a Raman Spectrometer to analyze the Raman scattering spectrum by focusing the groove channel after several rounds of washing and air-drying to eliminate the non-covalently bonded residuals. From Figure 5, we can see that the *p*MBA ethanol solution of higher concentration would enhance the Raman peaks of 1078 cm^−1^ and 1580 cm^−1^, which are the “fingerprint” spectra of *p*MBA due to the ν12 and ν8a planar vibrations of the benzene ring, respectively [45]. The result confirms that the gold layer in the groove captures the *p*MBA in the solution effectively and therefore reduces the refractive index of the liquid on a relatively large scale.

### 3.3. Contrast Response to the Non-Mercapto-Group Chemicals

**Figure 6:**
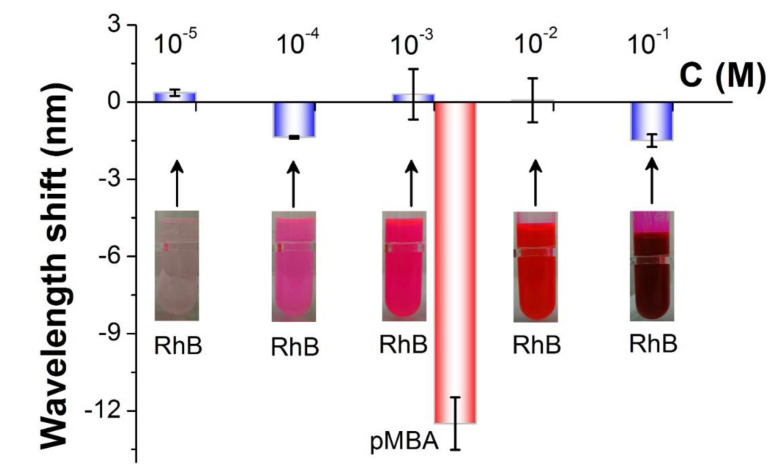
The response of the VIOLIN to different RhB ethanol solutions in comparison with the response to the moderate concentration of *p*MBA. “C” referred to “concentration”.

In order to prove the specificity of the VIOLIN, we employ the ethanol solution containing Rhodamine B, a kind of non-thiol chemical, in the experiment [46]. After pipetting the different concentrations of RhB ethanol solutions in the channel array, it can be seen in Figure 6 that the wavelength-shifting of the VIOLIN resonance exhibits irregular performance in spite of the concentration changing of the analytes. As well, the amounts of the wavelength shifts are less than 2 nm at different concentrations and much smaller in contrast with the *p*MBA test even at a moderate concentration of the solution (12 nm @10^−3^ M). The erratic and tiny response indicates the unaltered concentration of the solution in the channels due to that the molecular binding neither occurs at substates surface nor the fiber surface. The result manifests that the VIOLIN holds the specificity for targeting mercapto-group chemicals. 

### 3.4. Reproducibility of the VIOLIN Chemical Sensor

**Figure 7:**
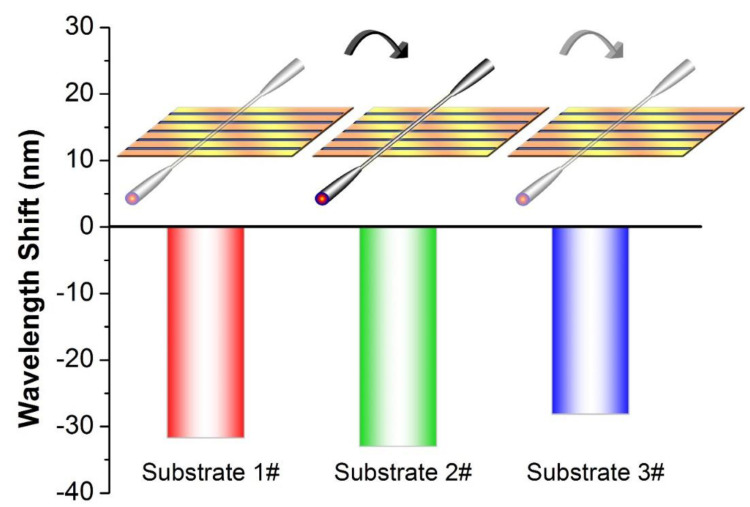
Response of the VIOLINs, using the substrates in a batch, with respect to 0.1 M *pMBA* solution. Inset: the scheme of substate-changing in the tests.

In the chemical or biological sensing area, recycling of the sensor, commonly applying “probe-target” molecularly specific binding mechanism, is often complicated due to the additional unbinding process. The VIOLIN configuration offers an excellent solution thanks to its unique superiority of flexible reconstruction of the components. The microfiber and substrate could be dissembled and assembled freely as though we are playing the bow and string pad from different real violin instruments. The convenience of the reproduction of the VIOLIN could be demonstrated via the changing of the substrates. The CO_2_ laser engraving technique provides high reproducibility in manufacturing and throughput to the substrates. Three of the substrates are randomly selected from a batch with the specified engraving parameters as mentioned above. The same microfiber is placed on those substrates in turns. The same concentration of 0.1 M *p*MBA ethanol solution is used throughout the tests. The results, shown in Figure 7, describe that the three VIOLINs present similar responses of blue-shift, −31 nm on average with a standard deviation of 2.5 nm, to the *p*MBA ethanol solution. Therefore, the detachable structure enables the proposed VIOLIN to revive by changing the substrates after an irreversible binding detection (*p*MBA molecules—Gold).

## 4. Conclusions

In summary, a highly reconfigurable and scalable long period fiber grating (VIOLIN) is demonstrated to be a fascinating analytical approach of the mercapto group chemicals. The liquids streaming in the periodic groove channels are devoted to the LPG as the modulating structure acting on the microfiber. The VIOLIN presents a high RI sensitivity over 2000 nm/RIU, allowing for perceiving the density change of the liquid caused by the precipitation of the solute. By gilding a gold layer, the VIOLIN is endowed with the capability of sensing mercapto group chemicals with high specificity compared with non-mercapto group chemicals. The response of the resonance purely depends on the alteration of the bulk refractive index as a result of the adsorption of the *pMBA* on the gold layer, which is confirmed by the Raman analysis in the groove channel. It is a paradigm shift to the traditional surface refractive index transducing regime, enabling an explicit and straightforward assay approach. Besides, the configuration offers high flexibility and reproducibility to the formation of LPG. The free detachability enables the VIOLIN to be reproduced by merely changing the substrate after an irreversible adsorption process without the laborious operation of applying a new fiber sensor. Future investigation should be focused on the surfactant modification in the groove to address the limitation of the aqueous fluidity caused by hydrophilia for expanding the application scenarios, especially for the case of the determination of biomolecules. Furthermore, programmable manipulation of the fluidic pipetting can be involved in tailoring the VIOLIN spectrum from several aspects, such as linewidth, chirping as well as phase-shifting, to provide more functionalities. Besides, several approaches of temperature compensating or simultaneous monitoring could be involved to overcome the temperature cross-sensitivity. It is predicted that the multiplexing of the VIOLIN could contribute to the temperature-RI simultaneous sensing by flowing different types of liquid with distinct thermo-optic coefficients. Overall, thanks to the high RI sensitivity, facility, reproducibility, and scalability, the sensor proposed in our scheme offers a new route to the field of chemical and biological sensing through catering to the need of high throughput test and commercialization.
